# Ionizing Radiation Induces Extracellular Trap Release from Macrophages

**DOI:** 10.3390/ijms27020993

**Published:** 2026-01-19

**Authors:** Yongchan Lee, Monowar Aziz, Ping Wang

**Affiliations:** 1 Center for Immunology and Inflammation, The Feinstein Institutes for Medical Research, 350 Community Dr., Manhasset, NY 11030, USA; 2 Departments of Surgery and Molecular Medicine, Zucker School of Medicine at Hofstra/Northwell, 350 Community Dr., Manhasset, NY 11030, USA

**Keywords:** macrophage, extracellular traps, METs, radiation, Gasdermin D, PAD2, PAD4

## Abstract

Macrophages are key innate immune cells in the host defense against pathogens. Ionizing radiation can impair macrophage functions such as phagocytosis and activate them, potentially exacerbating tissue injury. Macrophage extracellular traps (METs) are formed upon stimulation of macrophages with PAMPs or DAMPs. We hypothesized that macrophages exposed to ionizing radiation can release extracellular traps. Peritoneal macrophages were collected from C57BL/6 mice and subjected to 5 Gy radiation. We performed assays to detect METs, including the immunofluorescence of citrullination of histone H3 and cell-free DNA measurement in cell culture medium as well as cell death. The exposure of ionizing radiation killed a significant number of mouse peritoneal macrophages through pyroptosis, which was mediated by Gasdermin D (GSDMD). The onset of pyroptosis eventually caused METs by suicidal METosis via pyroptosis and vital METosis occurring in the cells surviving after exposure to radiation. We found that exposure of peritoneal macrophages to 5 Gy radiation significantly increased METosis, as revealed by increased levels of citrullinated histone H3 and an increased surface area of extracellular DNA surrounding the cells. We discovered that peptidyl arginine deiminase (PAD) 2 and 4 are required for peritoneal macrophages to generate extracellular traps in response to radiation exposure. Our data demonstrate that the ionizing radiation induces METs via the activation of GSDMD, and we confirmed the requirement of PADs for METosis after exposure to the ionizing radiation. Targeting METs may direct a new therapeutic strategy for mitigating radiation-induced tissue injury.

## 1. Introduction

High-energy ionizing radiation arises from both natural and human-made sources that can ionize atoms [1]. Life-threatening doses of ionizing radiation can arise from nuclear detonations, severe reactor or criticality accidents, unshielded high-activity sources in industry, radiotherapy misadministration, and extreme solar particle events affecting astronauts. Medical countermeasures are vital to rapidly reduce radiation injury, prevent complications, and improve survival [2]. Ionizing radiation disrupts the chemical bonds within molecules, which is highly damaging, especially in living tissues. Ionizing radiation primarily damages human health by disrupting cellular processes and DNA structure. This damage can lead to a range of effects, depending on the dose and exposure time. Severely damaged cells may undergo programmed cell death or necrosis [3]. When enough cells in a tissue or organ are damaged or die, the organ’s function is impaired, leading to symptoms like skin burns, gastrointestinal distress, bone marrow suppression, or organ failure [1]. Ionizing radiation severely suppresses the immune system by damaging and depleting highly radiosensitive immune cells, particularly lymphocytes and their bone marrow precursors [1]. This leads to a compromised ability to fight infections and an impaired capacity for tissue repair and immune surveillance.

Ionizing radiation significantly impacts macrophages by causing direct DNA damage and indirectly generating harmful free radicals, leading to their dysfunction or death. This results in impaired phagocytosis, altered cytokine production (often shifting towards a pro-inflammatory or pro-fibrotic profile), and modulated ability to present antigens [4,5,6].

Macrophage extracellular traps (METs) are web-like structures released by macrophages into the extracellular space, primarily composed of decondensed chromatin (DNA and associated histones) and various antimicrobial proteins and enzymes [7]. Like neutrophil extracellular traps (NETs), the main function of METs is to physically trap and concentrate pathogens (like bacteria, fungi, viruses, and parasites), preventing their dissemination and allowing the embedded proteins to neutralize or kill them [7]. MET formation has significant clinical implications in various diseases, including autoimmune diseases, cardiovascular disorders, cancer, infectious diseases, chronic inflammation conditions, and neurological disorders [8].

Peptidyl arginine deiminase (PAD) enzymes are required for initiating the formation of METs [9]. By chemically modifying histones through a process called citrullination, PADs cause the tightly packed chromatin to decondense and unravel. This released web of DNA then forms the structural backbone of the MET, which is expelled from the cell to trap pathogens. PAD2- and PAD4-mediated histone citrullination loosens chromatin. Intracellular granules help decondense DNA, which is ultimately expelled, either by suicidal membrane rupture or via vesicular export, in a vital process [10,11].

Gasdermin D (GSDMD) was discovered as the executioner of pyroptosis in 2015, and the release of IL-1β and IL-18 (and likely other IL-1 family members) was found to be dependent on GSDMD [12,13]. It was activated by inflammasome both dependently and independently [14]. The protein consists of an N-terminal pore-forming domain and a C-terminal inhibitory domain, with a cleavage site in the linker between the two domains at position D276 in mice and D275 in humans. GSDMD plays a crucial role in the release of METs [11,15]. Upon activation by extracellular cold-inducible RNA-binding protein (eCIRP), cleaved GSDMD forms large pores in the macrophage’s cell membrane. This pore formation leads to cell rupture, an inflammatory cell death process known as pyroptosis, which expels the pre-formed MET scaffold from the cell’s interior into the surrounding environment to combat pathogens.

Exposure to ionizing radiation resulted in extracellular trap formation from neutrophils [16]. However, any evidence of macrophage extracellular trap formation by ionizing radiation has not yet been reported. In this study, we discovered that macrophages release their DNA to the extracellular space in response to ionizing radiation. This extracellular DNA was modified in its histone protein with citrullination, and the process was dependent on the activation of GSDMD and PAD enzymatic activity. Thus, our findings shed light on how radiation exposure compromises macrophage function and identify a new therapeutic target to mitigate radiation-induced immune cell dysfunction.

## 2. Results

### 2.1. Ionizing Radiation Induces the Release of Macrophage Extracellular Traps (METs)

**Figure 1:**
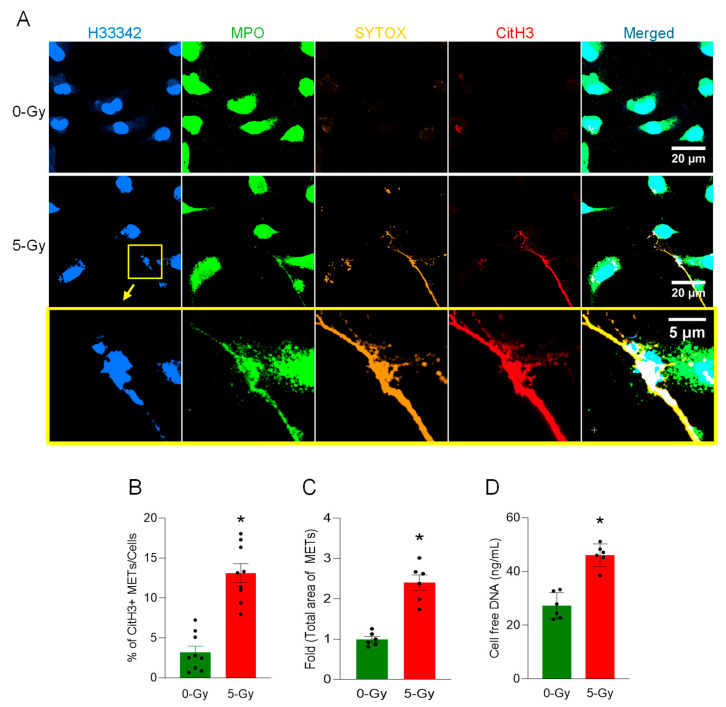
METs are released from peritoneal macrophages exposed to ionizing radiation. The release of METs from peritoneal cells exposed to radiation (5 Gy) was observed by confocal microscopy, (**A**) Confocal z-stack images were processed by maximum-intensity projection. The DNA was stained with Hoechst 33342 and SYTOX Orange in the cell culture media. Immunostaining for MPO and citrullinated Histone H3 was done after the fixation of the cells. The immunofluorescence microscopy shows that 5 Gy irradiation significantly increases the citrullination of histone H3, whereas no significant citrullinated histone H3 was detected in the control cells. Scale bar, 20 µm. The region in the yellow box in the middle panel is magnified to show the detail of the MET morphology; scale bar, 5 µm. The blown-up images of the yellow box show that CitH3 and MPO are colocalized in SYTOX Orange-positive extracellular DNA (bottom panel). (**B**) METs released in the culture media were quantified by image analysis. Student’s *t*-test: * *p* < 0.05 vs. 0 Gy control cells. Data were collected from three independent experiments, each with three image fields. (**C**) Based on the regions of interest detected in the immunofluorescence image of CitH3 and H33342, we calculated the total area of METs detected. Student’s *t*-test: * *p* < 0.05 vs. 0 Gy control cells. Data were collected from two independent experiments, each with three image fields. (**D**) Cell-free DNA in the culture media was quantified immediately after collecting the culture supernatant. Student’s *t*-test: * *p* < 0.05 vs. 0 Gy control cells. Data were collected from two independent experiments, each with three biological replicates.

To demonstrate the release of extracellular traps from macrophages following irradiation, irradiated macrophages were incubated for 24 h and fixed with 4% paraformaldehyde. We performed an immunofluorescence assay with anti-citrullinated histone H3 and anti-myeloperoxidase antibodies, which revealed MET formation in irradiated macrophages compared to the control (Figure 1A). The extracellular DNA released was detected with both antibodies. Selective staining of SYTOX Orange was obvious and exclusive on the extracellular DNA, as it is plasma membrane-impermeable. SYTOX Orange staining was only maintained during the cell culture and was washed away immediately prior to the fixation with paraformaldehyde. It was noted that there were cytoplasmic DNA puncta bright with all the colors in the viable cell (lower left corner of the middle panel image), which may indicate the vital release of extracellular traps [11]. The presence of extracellular DNA on the surface of cell culture vessels was significantly increased by irradiation. The level and the number of CitH3-positive extracellular DNA were measured by image processing of the immunofluorescence assay. The proportion of CitH3 over the total number of nuclei in the microscopic fields was significantly elevated upon the exposure of 5 Gy radiation from 3.2 to 13.1% (Figure 1B). The measurement of the absolute number of CitH3-positive DNA segments also showed a 3.5-fold increase compared to that of non-irradiated control cells. Total MET area with 5 Gy radiation was increased 2.4-fold compared to the control cell culture (Figure 1C). We also measured the concentration of cell-free DNA, which is free-floating DNA in the cell culture media. The concentration of cell-free DNA was increased to 170% by the irradiation (Figure 1D). These data show that radiation increases CitH3 and MPO, which are frequently used as MET markers.

### 2.2. Ionizing Radiation Induces Elevated Vesicular DNA and MET Release in Live Macrophages

**Figure 2:**
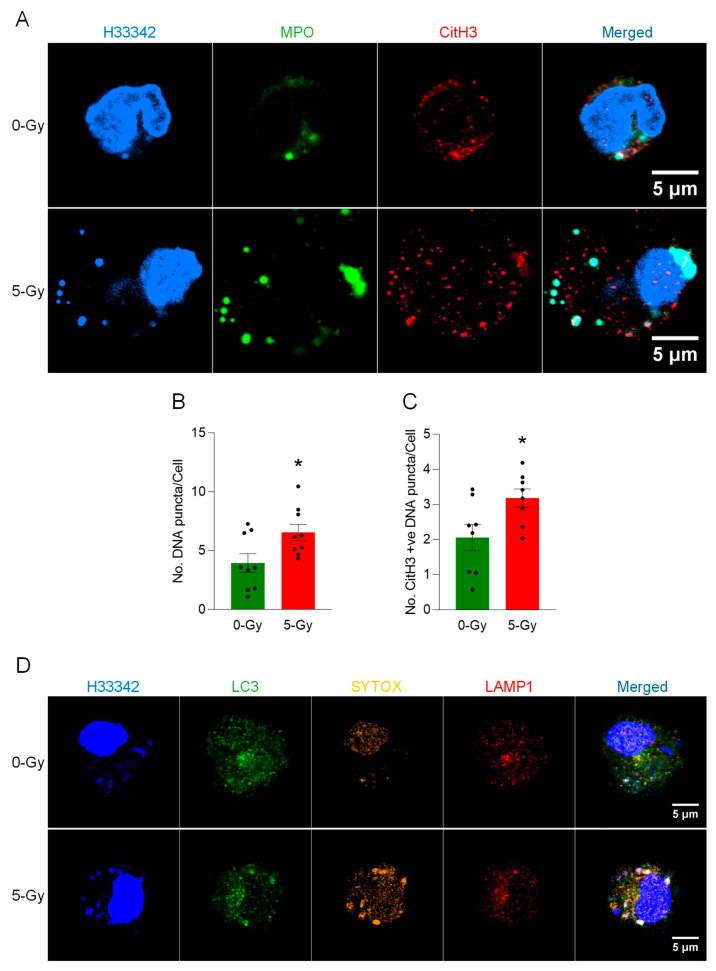
METs are released from live peritoneal macrophages. (**A**) The release of METs from live peritoneal macrophages exposed to ionizing radiation (5 Gy) was observed by confocal microscopy. The DNA was stained with Hoechst 33342 in the cell culture media. The immunostaining for MPO and citrullinated Histone H3 was performed after the fixation of the cells. The puncta in the cytoplasm were differentially stained with markers. Scale bar, 5 μm. (**B**) An increase of the number of DNA puncta in the cytosol of cells was observed in the cells exposed to 5 Gy radiation. Student’s *t*-test: * *p* < 0.05 vs. 0 Gy control cells. Data were collected from three independent experiments, each with three image fields. (**C**) The number of CitH3-positive DNA puncta was also increased after exposure to radiation. Student’s *t*-test: * *p* < 0.05 vs. 0 Gy control cells. Data were collected from three independent experiments, each with three image fields. (**D**) The DNA released from the nucleus was packaged as the LC3-positive vesicle and was incorporated into lysosomes together with LC3. The cells exposed to X-ray showed significant accumulation of LC3 in the DNA puncta. Scale bar, 5 μm.

Microscopic observation of peritoneal macrophages revealed the occurrence of vital MET release after irradiation. Immunofluorescence assay of cells exposed to 5 Gy radiation showed not only a massive structure of METs but also a significant increase in cytoplasmic DNA puncta in the cells surviving the irradiation (Figure 2A). DNA puncta were differentially stained with MPO and CitH3 or were unstained. The exposure of radiation induced an increase in the average number of intracellular DNA puncta. The average number of DNA puncta was increased to 166% by the irradiation (Figure 2B). CitH3 modification in each DNA punctum was also increased to 148% by the irradiation (Figure 2C). Further analysis showed that the intracellular DNA puncta were stained with anti-LC3 and anti-LAMP1 antibodies (Figure 2D). The differential staining of SYTOX, LC3, and LAMP1 support that the DNA released from the nucleus is processed in the endosomal compartment through autophagy machinery and destined for the lysosomal compartment.

### 2.3. Ionizing Radiation Induces Macrophage Cell Death

**Figure 3:**
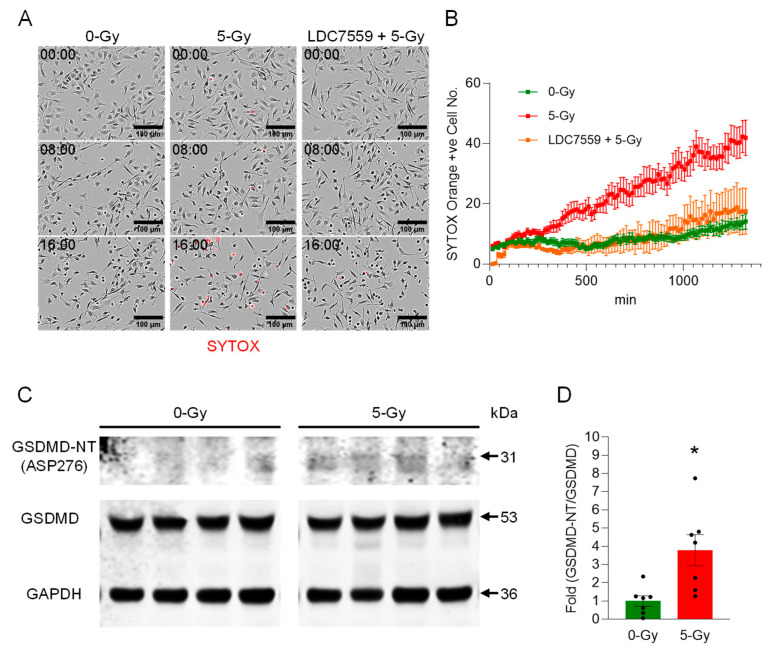
Ionizing radiation induces extracellular trap formation in peritoneal macrophages via Gasdermin D activation. (**A**) Peritoneal macrophages were exposed to 5 Gy radiation in the presence of SYTOX Orange (0.6 µM) and subjected to time-lapse live-cell imaging immediately after irradiation. The left panels show the cells without radiation exposure, and the middle panels show the cells exposed to 5 Gy radiation at different time points (0, 8, and 16 h after radiation exposure). The right panels show irradiated cells pretreated with LDC7559 (10 µM), which inhibits pore formation in activated Gasdermin D. Scale bar, 100 µm. (**B**) The radiation effect on macrophages in the live-cell imaging was quantified by counting the cells positive for SYTOX Orange. Images were acquired every 15 min over the time periods. The activation of Gasdermin D was confirmed by probing cleaved fragments of Gasdermin D. Data were collected from two independent experiments, each with four image fields. (**C**) Western blot analysis showed the increase of Gasdermin D-NT. (**D**) The fold ratio of GSDMD-NT/GSDMD was calculated. Student’s *t*-test: * *p* < 0.05 vs. 0 Gy control cells. Data were collected from two independent experiments, each with four and three biological replicates.

We assessed the onset of pyroptosis in peritoneal macrophages upon exposure to ionizing radiation and found it induced significant cell death. The membrane permeability of the peritoneal macrophages upon treatment with 5 Gy irradiation was monitored by a live imaging experiment with SYTOX Orange staining (Figure 3A). The live-cell imaging for 16 h showed that the treatment of 5 Gy of X-ray irradiation significantly increased the number of STOX Orange-positive dead cells, and the number of dead cells of 5 Gy exposed cells as compared to non-irradiated cells was 3-fold higher at the end of the recording (Figure 3B). This result shows that radiation exposure induces a rapid execution and significant extent of cell death in peritoneal macrophages.

### 2.4. Ionizing Radiation-Induced Membrane Rupture Is Mediated by Gasdermin D

Next, we aimed to study the cause of cell death due to radiation exposure. We treated irradiated cells with Gasdermin D pore-forming inhibitor LDC7559 (10 µM). The cells were exposed to radiation after 30 min incubation with LDC7559. The irradiation effect was apparently reversed by treatment with Gasdermin D inhibitor LDC7559 (Figure 3A,B). Our data showed that it could effectively maintain cell viability near the level of the non-irradiated control cell culture over time. This indicates that Gasdermin D is the key effector of cell death by irradiation. Thus, we speculated that the X-ray irradiation elicited the onset of pyroptosis, and subsequently DNA was released from the ruptured cells. The Western blot analysis of cleaved Gasdermin D showed an increased level of activated Gasdermin D N-terminal fragment (GSDMD-NT) in the whole-cell lysate samples of X-ray irradiated macrophages (Figure 3C and Supplementary Figure S1). The antibody specific to Asp276 of Gasdermin D was used to show that Gasdermin D was activated by the enzymatic cleavage of Caspase-1. Indeed, the cleavage product of Gasdermin D was increased 3.8-fold compared to non-irradiated control cells (Figure 3D). Our data show that Gasdermin D plays an important role in radiation-induced pyroptosis.

### 2.5. Ionizing Radiation Induces MET Formation via PAD2 and PAD4

**Figure 4:**
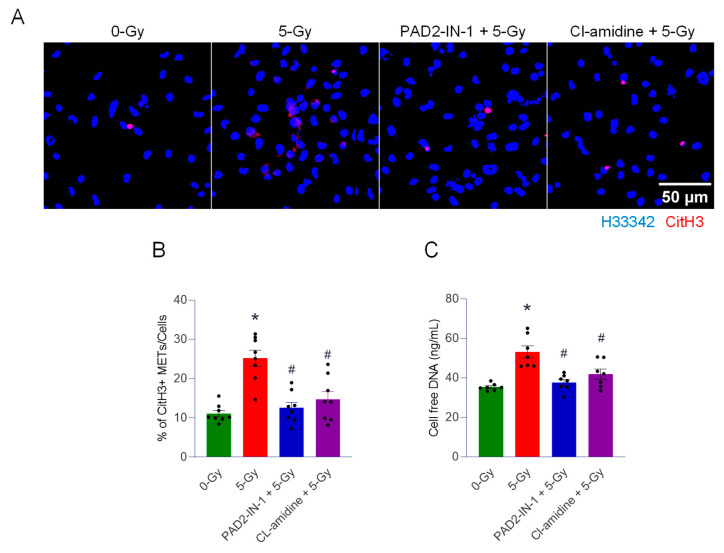
PADs are required for the formation of extracellular traps by ionizing radiation. We used two different enzyme inhibitors, PAD, PAD2-IN-1 (20 µM) and Cl-amidine (10 µM) (PAD4 inhibitor), to treat the cells exposed to 5 Gy radiation. (**A**) The effect of the inhibitors was assessed by immunofluorescence staining of the CitH3 antibody. DNA was stained with H33342, and CitH3 was detected with the anti-CitH3 antibody. The treatment of inhibitors was accompanied with 5 Gy radiation. (**B**) METs released were quantified by image analysis. One-way ANOVA: * *p* < 0.05 vs. 0 Gy control cells and ^#^ *p* < 0.05 vs. 5 Gy. Data were collected from two independent experiments, each with four biological replicates. (**C**) Cell-free DNA in the culture media was quantified immediately after collecting the culture supernatant. One-way ANOVA: * *p* < 0.05 vs. 0 Gy control cells and ^#^ *p* < 0.05 vs. 5 Gy. Data were collected from two independent experiments, each with three and four biological replicates.

To investigate the role of PAD2 and PAD4 in the cells exposed to 5 Gy radiation, we treated PAD2-IN-1 (20 µM) and cl-amidine (PAD4 inhibitor) (10 µM). The cells were exposed to radiation after 30 min incubation of the inhibitor. First, we observed the citrullination of histone H3 in response to irradiation. The immunofluorescence of the irradiated cells with anti-CitH3 antibody was assessed. The treatment of both inhibitors could effectively abolish the formation of METs by irradiation (Figure 4A). CitH3-positive METs per cell was quantified, and it was significantly increased, to 28%, when the cells were exposed to 5 Gy irradiation (Figure 4B). Control cells showed 11%. Treatment of PAD2-IN-1 and cl-amidine brought it down to significantly low levels of 12% and 16%, respectively. Cell-free DNA measurement further supported the effect of both PAD inhibitors on the formation of extracellular traps by irradiation (Figure 4C). Radiation elicited significantly higher levels of DNA release in the culture medium compared to that of control cells. Treatment with PAD2 and PAD4 inhibitors significantly decreased the level of cell-free DNA to the basal level (Figure 4C). Our data clearly show that PADs are required for radiation-induced MET formation.

### 2.6. Ionizing Radiation Induces METs via Gasdermin D Activation

**Figure 5:**
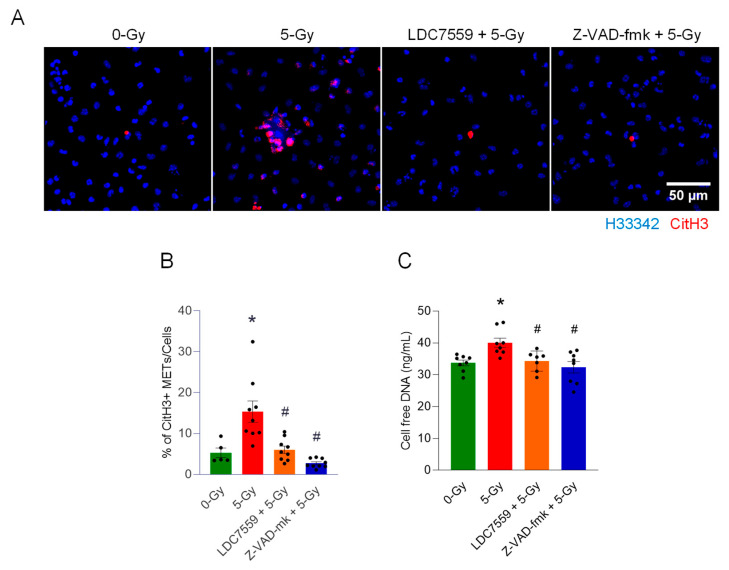
Gasdermin D activation is required for the formation of extracellular traps by ionizing radiation. The role of Gasdermin D in extracellular trap formation was assessed by treatment with the inhibitor of Gasdermin D pore formation, LDC7559 (10 µM), and the pan-caspase inhibitor Z-VAD-fmk (20 µM). Inhibitors were applied to the cells exposed to 5 Gy radiation. (**A**) The effect of the inhibitors was assessed by immunofluorescence staining of the CitH3 antibody. DNA was stained with H33342, and CitH3 was detected with anti-CitH3 antibody. The treatment of inhibitors was accompanied with 5 Gy radiation. (**B**) METs released were quantified by image analysis. One-way ANOVA: * *p* < 0.05 vs. 0 Gy control cells and ^#^ *p* < 0.05 vs. 5 Gy. Data were collected from two independent experiments, each with five image fields. (**C**) Cell-free DNA in the culture media was quantified immediately after collecting the culture supernatant. One-way ANOVA: * *p* < 0.05 vs. 0 Gy control cells and ^#^ *p* < 0.05 vs. 5 Gy. Data were collected from two independent experiments, each with three and four biological replicates.

To further identify the contribution of pyroptotic cell death to the release of macrophage extracellular traps, we performed an immunofluorescence assay with the antibody against citrullinated histone H3 (CitH3) in peritoneal macrophages treated with 5 Gy X-ray irradiation with or without inhibitors, including LDC7559 (10 µM) (Gasdermin D pore formation inhibitor) and Z-VAD-fmk (20 µM) (pan-caspase inhibitor). Cells were exposed to radiation after 30 min incubation of the inhibitor (Figure 5A). Irradiated macrophages committed pyroptotic cell death and released extracellular traps. CitH3-positive cell-free DNA was frequently observed with the cluster of dead cells, which have nuclei densely stained with Hoechst 33342 and the CitH3 antibody. The formation of extracellular traps was significantly reduced by the inhibition of Gasdermin D pore formation. The inhibition of Gasdermin D pore formation with small molecule LDC7559 effectively decreases overall CitH3 staining. Clusters of ETs and dead cells were also scarce. Pan-caspase inhibitor Z-VAD-fmk treatment showed even stronger inhibition of CitH3 modification than that of LDC7559 in the cells. The measurement of CitH3 METs and cell-free DNA showed the effectiveness of both inhibitors. Irradiation increased the level of CitH3 METs per cell to 15.3%, 3-fold higher than control cells (5.3%). The treatment of macrophages with Gasdermin D pore formation inhibitor, LDC7559, significantly decreased the formation of METs following radiation exposure, to 6% (Figure 5B). Cell-free DNA measurement also showed results consistent with the CitH3 immunofluorescence assay (Figure 5C). These findings indicate that blocking the pore formation of Gasdermin D alone is sufficient to effectively prevent the cells exposed to radiation from releasing MET formations.

## 3. Discussion

**Figure 6:**
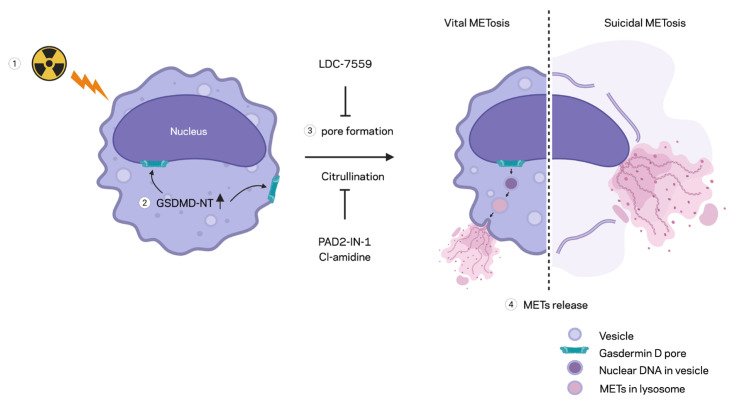
Schematic summary of MET formation by radiation. Radiation-induced MET formation requires PAD enzyme activity, which facilitates histone H3 citrullination for chromatin decondensation. Ionizing radiation also triggers GSDMD activation, leading to the formation of a membrane pore that facilitates MET expulsion. Inhibition by PAD inhibitors or GSDMD inhibitors reduces MET formation by preventing histone H3 citrullination and GSDMD pore formation, respectively, following radiation exposure.

In this study, we demonstrated the formation of extracellular traps in mouse peritoneal macrophages exposed to ionizing radiation. The METs induced by ionizing radiation carried components like citrullination of histone and MPO, as they were observed in different circumstances and routes of release, including suicidal and vesicular transport. Employing a GSDMD inhibitor and PAD inhibitors, we showed the involvement of GSDMD in MET formation and, furthermore, the indispensable role of PADs (Figure 6). Our study showed radiation exposure induced pyroptotic cell death in macrophages and the release of extracellular traps. Radiation modulates macrophage phenotype and function, often shifting cytokine profiles and surface marker expression. It can impair phagocytic capacity while altering the secretion of inflammatory mediators [6,17]. At higher doses, radiation increases macrophage cell death, reducing tissue homeostatic support. Ionizing radiation generates DNA damage and oxidative stress, which can activate danger-sensing pathways in exposed cells. Radiation-induced damage engages priming inflammasome components such as NLRP3 [18]. Activation of inflammasomes leads to caspase-1 (or caspase-4/5/11 in the noncanonical route) cleavage of GSDMD, creating pore-forming N-terminal fragments. GSDMD pores perforate the plasma membrane, causing ionic fluxes, cell swelling, and the release of IL-1β and IL-18. The culmination is lytic cell death (pyroptosis), which amplifies inflammation and can exacerbate radiation tissue injury. Both suicidal and vital METosis were observed in this study. Suicidal METosis is a trap-forming process in which a macrophage undergoes terminal cell death, characterized by chromatin decondensation, membrane rupture, and the release of DNA strands decorated with granule enzymes into the extracellular space [7,15]. Vital METosis, in contrast, allows macrophages to expel extracellular traps while preserving plasma membrane integrity and continued viability, via vesicular export of DNA [11].

We also showed the involvement of PAD2 and 4 in disseminating the extracellular traps of macrophages exposed to radiation. We employed two enzyme inhibitors, PAD2-IN-1 and Cl-amidine. Both inhibitors were characterized, as they inhibit the isoforms of PAD differentially. PAD2-IN-1 is known to preferentially affect PAD2 95-fold higher than PAD4, whereas Cl-amidine has broad specificity for PADs, including PAD1, PAD3, and PAD4. Both inhibitors effectively inhibited MET formation in the macrophages exposed to the ionizing radiation. To evaluate the activity of PADs, we probed the citrullination of histone H3 protein, which has been frequently used as a marker of extracellular trap detection [19]. Protein citrullination is a post-translational modification (PTM) that is catalyzed by the PAD family of enzymes. Among the isoforms of PAD, PAD2 and PAD4 were identified as required for macrophages generating extracellular traps [9]. In our study, the treatment of both inhibitors decreased not only the citrullination of histone but also the cell-free DNA measurement, which includes the levels of LDC7559 and Z-VAD-fmk. This reminds us that the inhibition of PADs has profound effects on cellular physiology, including cell death, inflammation, and DNA/RNA processing pathways, in macrophages [20,21].

Radiation-induced extracellular traps have been observed in neutrophils [22]. NETs generated by ionizing radiation exacerbate subsequent infection and inflammation after exposure to ionizing radiation. It was demonstrated that NETs were generated with the activation of the triggering receptor expressed on myeloid cells-1. NET is also considered as an important prognostic implication due to NET’s association with metastases, therapeutic resistance, and immunosuppression [16,23]. Here, we showed that macrophages could release extracellular traps as low as 5 Gy in vitro. Though we showed the requirement of GSDMD and PADs in the formation of MET by radiation, we still need to identify how the MET formation is controlled by upstream signaling in detail. Even though we successfully demonstrated the MET formation in the cells exposed to a single radiation dose of 5 Gy in vitro, due to the lack of systemic investigation, which was beyond the scope of current study, on the role of macrophage extracellular traps in response to ionizing radiation, there remain open questions on what dose is sufficient to induce MET formation and cause tissue injury as mediated by the METs produced. Further systemic investigations in the future are anticipated. These will provide invaluable information about susceptibility to the injury caused by radiation-induced METs in different organs like the peritoneum, spleen, and others due to subsequent infection and inflammation.

## 4. Materials and Methods

### 4.1. Reagents

Hoechst 33342 (H33342) (cat. no. R37605) and SYTOX Orange (cat. no. S11368) were purchased from Thermo Fisher Scientific (Waltham, MA, USA). Antibodies against cleaved Gasdermin D (cat. no. 10137), Gasdermin D (cat. no. 39754), and LC3B (cat. no. 2775) were purchased from Cell Signaling Technology (Danvers, MA, USA). Anti-GAPDH antibody (cat. No. 60004-1-Ig) was purchased from Proteintech (Rosemont, IL, USA). Anti-MPO-FITC (cat. no. ab90812), Alexa Fluor 647 anti-LAMP1 antibody (cat. No. ab237307), and anti-histone H3 (citrulline R2 + R8 + R17) antibody (cat. no. ab281584) were purchased from Abcam (Waltham, MA, USA). Z-VAD-fmk (final concentration, 20 μM) was purchased from Sellekchem, Houston TX, USA (cat no. S7023). LDC7559 (final concentration, 10 μM), Cl-amidine (Final concentration, 10 μM), and PAD2-IN-1 (AFM32a hydrochloride) (final concentration, 20 μM) were purchased from MedChemExpress (Monmouth Junction, NJ, USA). Inhibitors were all dissolved in dimethyl sulfoxide (DMSO). Inhibitor treatment was performed 30 min prior to the radiation treatment. DMSO (final concentration, 0.5% *v*/*v*) was used as a control. Inhibitor treatment was continued for 24 h.

### 4.2. Experimental Animals

C57BL/6 wild-type male mice (8 to 12 weeks) were purchased from the Jackson Laboratory (Bar Harbor, ME, USA). Mice were housed in a temperature-controlled environment under a 12 h light cycle with ad libitum access to standard rodent chow and water. All animal experiments were performed in accordance with the guidelines for using experimental animals by the National Institutes of Health. All study procedures were approved by the Institutional Animal Care and Use Committees of the Feinstein Institutes for Medical Research.

### 4.3. Cell Culture

Mouse peritoneal lavage was performed to collect peritoneal cells from unstimulated normal mice [24]. The mouse peritoneum was flushed with 10 mL cold PBS supplemented with 2% fetal bovine serum. For each experiment, peritoneal exudate cells from 8–12 mice were collected by centrifuge 400× *g* for 10 min at 4 °C and pooled. Cells were seeded in a cell culture vessel and incubated overnight. Cells were maintained in RPMI media with fetal bovine serum (10%) and penicillin–streptomycin. In 12-well plate cells, 12 mL of peritoneal cell suspension from 6 mice was aliquoted to each well. For 6-well plate cells, 12 mL of peritoneal cell suspension from 6 mice was aliquoted to each well. The culture vessel was then washed 3 times with fresh cell culture medium after overnight incubation. The inhibitor was applied 30 min before the radiation exposure. Cell culture was maintained for 24 h after radiation treatment. Samples were collected and processed for further analysis.

### 4.4. X-Ray Irradiation

Peritoneal macrophages were subjected to a single dose of 5 Gy with an X-ray irradiation system, X-Rad320 (Precision X-Ray Inc., Madison, CT, USA). Cells were incubated for 24 h after irradiation.

### 4.5. Time-Lapse Microscopy

Time-lapse microscopy was performed with an Incucyte live imaging system (Sartorius, Göttingen, Germany). Time-lapse images were recorded for 16 h at intervals of 15 min. Transmit light and red channel were recorded for detecting SYTOX Orange dead-cell staining. For live-cell imaging, glass-bottom culture plates were used. The 35 mm glass-bottom culture dish (part no.: P35G-1.5-20-C) and 6-well (part no.: P06G-1.5-20-F) and 12-well (part no.: P12G-1.5-14-F) plates were purchased from MatTek Life Sciences (Ashland, MA, USA).

### 4.6. Immunofluorescence

Cells were incubated for 24 h after radiation exposure, fixed for 15 min at room temperature with paraformaldehyde (4%), and washed 3 times with PBS. The fixed cells were immediately blocked with blocking buffer (5% BSA and 100 mM glycine in PBS) for 2 h, followed by the incubation of the primary antibody overnight at 4 °C. Antibody was diluted in the blocking buffer according to the manufacturer’s recommendations. The cells were then washed 3 times with PBS and incubated with fluorescence tagged secondary antibody for 2 h. After washing the cells 3 times with PBS, the immunofluorescence specimen was prepared by adding prolong gold antifade (cat. no.: P36934, Thermo Fisher Scientific).

### 4.7. Confocal Microscopy

Images with high resolution were obtained using the Zeiss Axio Observer.Z1/7 equipped with an LSM900 Airyscan confocal system (White Plains, NY, USA). Images of cells were acquired as a z-stack with a Plan-Apochromat 63×/1.40 Oil DIC M27 objective lens. The SR-4Y fast Airyscan acquisition mode and 4× averaging was used. The z-stack images obtained by Airyscan were merged and combined by FIJI ImageJ (Version 1.54p, National Institute of Health, USA) [25].

### 4.8. Quantitative Image Analysis

Low-magnification confocal images with immunofluorescence staining were acquired and analyzed by FIJI ImageJ. The Plan-Apochromat 10X/0.45 M27 objective was used for imaging. Nuclear counting was performed with Hoechst 33342 image. Citrullinated Histone H3 (CitH3) quantification was conducted using immunofluorescence staining with anti-citrullinated Histone H3 antibody. To quantify the CitH3 signal only in the MET area, the CitH3 signal in the nuclear region was removed by applying the ROI of the nuclear region. Then, the MET region was counted by generating a binary mask with thresholding command. The resulting MET count was normalized by the number of nuclei in the field.

### 4.9. Western Blotting

The cell-free lysate of macrophages was prepared by incubating RIPA buffer in each sample. The lysis buffer was supplemented with Sodium Orthovanadate (2 mM), phenylmethylsulfonyl fluoride (PMSF) (0.2 mM), and cOmplet mini-protease inhibitor cocktail from Roche (cat. No.: 11836153001; Millipore, Sigma, St. Louis, MO, USA). The protein content of the lysate was determined with a protein assay reagent (cat. no.: 5000002, Bio-Rad, Hercules, CA, USA). Each sample was separated by SDS-PAGE using 4–12% Bis-Tris gel (Invitrogen, NP0322, Thermo Fisher Scientific). Proteins in the gel were then transferred to a nitrocellulose membrane by the X cell II blot module. The membrane was incubated with primary antibody overnight at 4 °C, followed by the incubation of Odyssey secondary antibody (cat. no.: 926-32211 or 926-68070, Lycor Biosciences, Lincoln, NE, USA). The detection and quantification of Western blot was performed by the Odyssey CLx imaging system (Lycor Biosciences).

### 4.10. Cell-Free DNA Quantification

All cell samples were prepared in 12-well culture plates and maintained with 1 mL cell culture medium per each well in the plate. Cell culture media samples were collected at 24 h after radiation exposure by pipetting. The bottom of each well of the plate was flushed 5 times. EDTA (10 mM) was added to the sample to prevent DNase enzyme activity in the media. The culture media were subjected to centrifuge, 400× *g* for 10 min at 4 °C. The cell pellets were discarded, and the supernatants were immediately used for DNA measurement with a picogreen DNA quantification kit (Thermo Fisher Scientific).

### 4.11. Statistical Analysis

Data represented in the figures are expressed as mean ± SEM. The two-tailed Student’s *t*-test was used for two-group comparison, and one-way ANOVA with the post hoc Tukey test was used for multiple-group comparison. A statistical significance threshold was considered for *p* ≤ 0.05 between study groups. Data analyses and graph preparation were carried out using GraphPad Prism (Version 10.2.0 (392)) graphing and statistical software (GraphPad Software, San Diego, CA, USA).

## 5. Conclusions

We discovered that ionizing radiation induces MET release. GSDMD activation was required for the pyroptotic death and resulting MET release. PADs served a key role in radiation-induced MET formation. Therefore, targeting Gasdermin D and PADs to regulate METs potentially offers future therapeutic strategies for alleviating tissue damage after radiation exposure.

## Data Availability

The original contributions presented in the study are included in the article/Supplementary Materials. Further inquiries can be directed to the corresponding authors.

## Supplementary Materials

The supporting information can be downloaded at: https://www.mdpi.com/article/10.3390/ijms27020993/s1.

## Abbreviations

ANOVA: analysis of variance, CitH3: citrullinated histone H3, DAMP: damage-associated molecular pattern, GAPDH: glyceraldehyde-3-phosphate dehydrogenase, GSDMD: gasdermin D, GSDMD-NT: gasdermin D n-terminal fragment, MET: macrophage extracellular trap, MPO: myeloperoxidase, NET: neutrophil extracellular trap, NLRP3: NLR family pyrin domain-containing 3, PAD: peptidyl arginine deiminase, PAMP: pathogen-associated molecular pattern, SEM: standard error of the mean.
