# Supplementary material for: Ionizing Radiation Induces Extracellular Trap Release from Macrophages
Source: Int J Mol Sci. 2026 Jan 19;27(2):993. doi: 10.3390/ijms27020993 (PMC12841997; doi:10.3390/ijms27020993)
Supplement: Supplementary file 1 [file ijms-27-00993-s001.zip › ijms-4076777-supplementary.pdf]

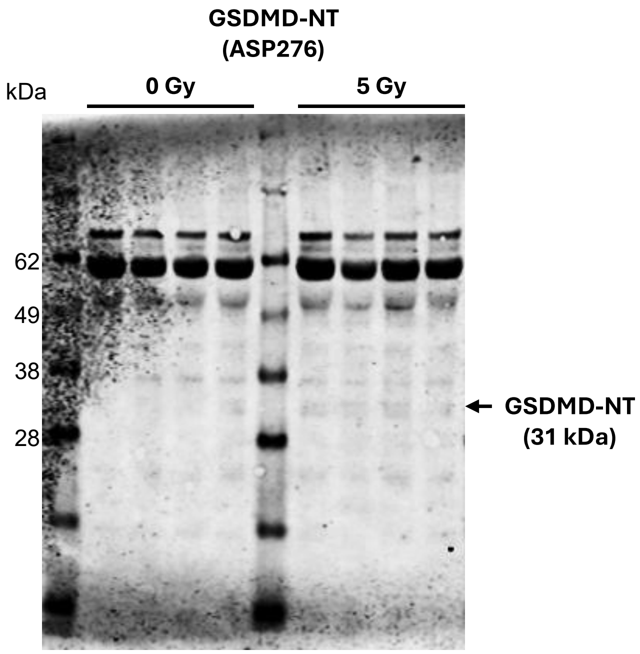

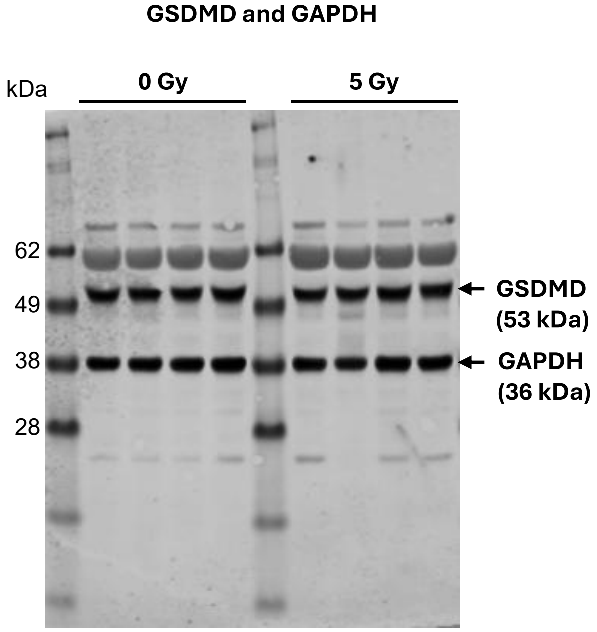

Supplementary Figure S1. Full gel image of Western blot. Gasdermin D activation was probed by anti-Asp276 GSDMD n-terminal fragment Abs, anti-GSDMD full length antibody, and anti-GAPDH Abs. Anti-Asp276 GSDMD n-terminal fragment Abs was probed first, and the nitrocellulose membrane was stripped and probed with the rest antibodies at the same time.
